# Boosting secretion of starch-converting enzymes from *Priestia koreensis* HL12 and its application in non-thermal cassava pulp saccharification process for maltooligosaccharides synthesis

**DOI:** 10.1186/s40643-025-00872-x

**Published:** 2025-04-21

**Authors:** Daran Prongjit, Benjarat Bunterngsook, Wuttichai Mhuantong, Katesuda Aiewviriyasakul, Wipawee Sritusnee, Hataikarn Lekakarn

**Affiliations:** 1 Department of Biotechnology, Faculty of Science and Technology, Thammasat University, Rangsit Campus, Khlong Nueang, Khlong Luang, 12120 Pathum Thani Thailand; 2 Enzyme Technology Research Team, Biorefinery Technology and Bioproduct Research Group, National Center for Genetic Engineering and Biotechnology, 113 Thailand Science Park, Phahonyothin Road, Khlong Nueang, Khlong Luang, 12120 Pathum Thani Thailand

**Keywords:** Biorefinery, Genomics, Proteomics, *Priestia koreensis*, Saccharification, Starch-converting enzyme

## Abstract

Exploring novel amylolytic enzymes and understanding their biochemical properties are essential for advancing starch-based industries. This study focused on identifying the effective extracellular enzymes produced by *Priestia koreensis* HL12, a novel starch-converting bacterium, through a combined proteomics and genomics approach. Genome annotation revealed 82 genes belonging to various CAZyme families. Among the ten media tested for amylolytic enzyme production, raw cassava pulp was identified as the most effective carbon source, yielding a remarkable starch-degrading activity of 452.6 ± 2.944 U/mg protein against cassava starch. The extracellular enzymes demonstrated significant potential for high-solid enzymatic hydrolysis of raw cassava pulp (5% w/v), achieving a conversion rate of 72% (719.1 mg/g biomass) at 45 °C, pH 5.0 for 24 h of hydrolysis. Proteomics analysis provided insights into the specific enzymes responsible for efficient starch breakdown and modification. These findings position *P. koreensis* HL12 as a highly effective bacterium for cellulase/xylanase-free amylase production. This work not only highlights the unique enzymatic profile of *P. koreensis* HL12 but also emphasizes its significant role in optimizing starch bioconversion processes, ultimately contributing to the development of more sustainable biorefineries.

**Supplementary Information:**

The online version contains supplementary material available at 10.1186/s40643-025-00872-x.

## Introduction

Starches are carbohydrates that play crucial roles in nutrition for both humans and animals. They are also versatile components in various starch-related industries. Starch consists of two polysaccharides: (i) amylose and (ii) amylopectin. Amylose is a linear chain of glucose molecules linked by α-1,4-glycosidic linkages, while amylopectin is a highly branched polymer of glucose units, featuring α-1,4-glycosidic linkages in the linear backbones and α-1,6-glycosidic linkages at the branching points. Generally, starches contain amylopectin as the major component with approximately 4% to 5% branching points that can hinder enzymatic action. Starch is produced by various plants, including corn, cassava, potato, and rice, each contributing distinct characteristics and properties based on their botanical origin (Seung 2020).

As the world's largest cassava exporter, Thailand generates a substantial amount of cassava processing waste, particularly cassava pulp (CP) averaging 5.15–7.30 million metric tons annually (Ghimire et al. 2015; Trakulvichean et al. 2017). This waste has a negative impact on environmental issues, particularly air pollution and waste management. Cassava pulp is primarily composed of 51% starch granules, making its high residual starch content a unique feedstock for biorefineries (Bunterngsook et al. 2017). Cassava pulp treatment involves various methods to enhance its utilization, including acid and alkaline hydrolysis, steam explosion and fermentation for animal feed and biogas production. Fermentation is essential in biorefinery for the bioconversion of food waste and biomass into valuable products, including functional ingredients, enzymes, biofuels, and biochemicals. This process promotes waste valorization, sustainability, and economic benefits by transforming low-cost feedstock into high-value products (Siddiqui et al. 2023, 2025). The starch-based biorefinery relies on enzymatic approaches to produce various products such as glucose, maltose syrup, maltodextrin, cyclodextrin, and maltooligosaccharides.

Maltooligosaccharides (MOS) are homooligosaccharides composed 3–10 of glucose unit, which are internally linked α-1,4-glycosidic linkages. MOS have been investigated for their prebiotic and anti-plant pathogen properties, which have potential applications as a functional ingredient in the pharmaceutical, nutraceutical, and agricultural industries (Bláhová et al. 2023; Jang et al. 2020; Li et al. 2021). In addition to their biological functions, the physicochemical properties of MOS, such as solubility, stability, mild sweetness, low bulk density, ability to inhibit crystallization of sucrose, and low osmolality, contribute to their extensive applications in the food and beverage industry. These include their use as an antistaling agent, low-calorie sweeteners, moisture regulators of food, and enhancers of beer foam stability and quality (Bláhová et al. 2023; Chen et al. 2015; Nakakuki 2002). Currently, the efficient production of MOS relies on advanced biotechnological approaches that have replaced traditional chemical processes. These methods utilize specific starch-degrading and modifying enzymes from microbes, such as α-amylase, maltooligosaccharide-forming amylase, and glycosyltransferase (Auiewiriyanukul et al. 2018; Bláhová et al. 2023; Lekakarn et al. 2022; Prongjit et al. 2022; Zhu et al. 2018). Consequently, high-performance starch-degrading and converting enzymes play vital roles in bioprocessing in the starch industry. The growing focus on sustainable development goals (SDGs) and minimizing environmental impact is driving the expansion of starch-based biorefineries, thereby increasing the demand for efficient amylolytic bacteria and starch-degrading enzymes.

Starch-degrading enzymes, also known as amylolytic enzymes, efficiently catalyze the hydrolysis of α-1, 4 glycosidic linkages and α-1, 6 glycosidic linkages in starch and glycogen. According to the sequence similarity classification of Carbohydrate-Active Enzymes (CAZymes) (http://www.cazy.org/), most amylolytic enzymes belong to glycoside hydrolase (GH) classes, particularly glycoside hydrolase family 13 (GH13). Additionally, some of these enzymes are classified as glycosyl transferases (GT), polysaccharide lyases, auxiliary activities family 13 (AA13), and lytic polysaccharide monooxygenases (LPMOs) (CAZymes) (http://www.cazy.org/). Enzymes belonging to the GH families hydrolyze glycosidic bonds between two or more carbohydrate types or between carbohydrates and non-carbohydrate components. The CAZymes database lists 172 GH families. Among these, the families considered as relevant to starch-degrading enzymes include GH3, GH13, GH14, GH15, GH31, GH57, GH119, and GH126, as well as auxiliary activities family 13 (AA13) and glycosyl transfer family 35 (GT35) (Janecek and Svensson 2022; Møller and Svensson 2016).

*Priestia* has the potential to be considered a Generally Recognized as Safe (GRAS) strain with desirable characteristics for use as an enzyme-producing bacterium. *Priestia* species were previously classified within the *Bacillus* genus, which is widely recognized as an excellent source of enzyme producers in biotechnology, known for their high protein production and secretory capacity. Furthermore, these bacteria are suitable for large-scale fermentation using low-cost culture media. Notably, several *Bacillus* species, such as *Bacillus licheniformis, Bacillus coagulans,* and *Bacillus subtilis*, are classified as GRAS strains and are commercially used in the food sector (FDA., USA). *Bacillus* species serve as effective cell factories for the production of various starch-degrading enzymes, including pullulanase (EC 3.2.1.41), glucoamylase (EC 3.2.1.3), ꞵ-amylase (EC 3.2.1.2), α-amylase (EC 3.2.1.1), and cyclodextrin glucanotransferase (EC 2.4.1.19). These enzymes are widely used in starch processing within the food industry (Farias et al. 2021). Starch-degrading enzymes from *Bacillus* species exhibit advantageous characteristics such as thermostability, stability in acidic or alkaline conditions, and activity at both low and high temperatures (Farias et al. 2021; Gu et al. 2018).

The exploration and study of starch-degrading enzymes is critical for acquiring deeper insights into the biological decomposition of starch-containing materials, which is essential for developing starch-based biorefineries. This research represents the first study to explore and identify effective extracellular starch-converting enzymes produced by the amylolytic bacterium *P. koreensis* HL12, employing a combined genomics and proteomics approach. This pioneering work lays the foundation for harnessing the enzymatic potential of *P. koreensis* HL12 in bioprocessing applications.

## Materials and methods

### Bacterial strain and chemicals

An amylolytic bacterium, *P. koreensis* HL12, was isolated from soil attached to sago palm root (*Cycas revoluta*) (Lekakarn et al. 2022). Cassava starch was purchased from Thai Wah Food Products Public Co., Ltd (Bangkok, Thailand). Cassava pulp was kindly provided by Chorchaiwat Industry Co. Ltd. (Bangkok, Thailand). All chemicals and reagents were of analytical grade and obtained from major chemical suppliers (Sigma, Merck, and Fluka).

### Genomic analysis

The genome sequencing of *P. koreensis* HL12 was carried out using the Illumina 150 PE platform (Novogene, China) and annotated according to the bioinformatics pipeline reported in Lekakarn et al. 2024. The putative genes related to the carbohydrate-active enzyme (CAZyme) family were predicted by dbCAN2 (Zhang et al. 2018).

### Media optimization for extracellular starch-converting enzymes production

Luria–Bertani (LB) broth and minimal medium without starch inducers were used as basal media. Minimal media supplemented with 1% (w/v) of different starch carbon sources were used as induction media for starch-degrading enzyme productions. Various starches and starch-type polysaccharides used as inducers were gelatinized starches (soluble starch, cassava starch, and cassava pulp), raw starches (raw cassava starch and raw cassava pulp), maltose, maltodextrin DE 4–7 (15.7–27.6 oligomers), and maltodextrin DE 13–17 (6.4–8.4 oligomers). The 1% (w/v) of inducers were added into minimal media and sterilized by autoclaving at 121 °C for 15 min. The raw starch inducers were separately sterilized and added to minimal media before inoculation to avoid the gelatinization process and moisture from autoclaving. *P. koreensis* HL12 was streaked on LB agar and incubated at 30 °C for 48–72 h. A single colony was inoculated into LB broth for starter preparation. The starter culture was incubated in an orbital shaker at 30 °C and 200 rpm for 24 h. Afterward, 1% (w/v) of the starter culture was transferred into 250 mL flasks containing 50 mL of media and incubated at 30 °C and 200 rpm for 72 h. Five mL of cultures were collected every 12 h until 72 h for cell growth and amylolytic enzyme monitoring. Cell growth was monitored using a spectrophotometer with absorbance at 600 nm. Collected samples were centrifuged at 8,000 × *g* for 5 min to separate secreted enzymes. The starch-degrading enzyme activity and protein profiling of secreted enzymes in supernatants were determined. Among the 12 different media, media supplemented with inducer showing significant cell growth and high activity of starch-degrading enzyme were selected for enzyme production.

### Enzyme activity determination

The amylolytic activity was determined according to the hydrolysis reaction of extracellular enzymes against soluble starch and cassava starch. The reaction mixture contained 1% (w/v) substrate dissolved in 50 mM sodium acetate buffer pH 5.0. The mixture was pre-incubated at 50 °C for 10 min, then an extracellular crude enzyme with an appropriate concentration was added to the reaction and incubated at 50 °C for 10 min. Cellulase, xylanase, and pectinase activity were tested using carboxymethyl cellulose (CMC), beechwood xylan, and polygalacturonic acid as substrates, respectively. The reaction was stopped by adding DNS solution. The reducing sugar determination was carried out following the DNS colorimetric method (Miller 1959). One unit of enzyme was defined as the amount of enzyme that released 1 µmole of product per minute. To identify the optimal condition for effective amylase activity, the effects of pH and temperature on amylase activity of crude extracellular enzymes were investigated. The effect of pH was measured under different pH ranges in various buffers: 50 mM sodium citrate buffer pH 3.0–6.0, 50 mM sodium acetate buffer pH 4.0–5.0, 50 mM sodium phosphate buffer pH 6.0–8.0, and 50 mM Tris–HCl pH 8.0–9.0. The reaction was incubated at 50 °C for 10 min. The effect of temperature on amylase activity was evaluated at temperatures ranging from 20 to 90 °C in 50 mM citrate buffer pH 5.0 for 10 min.

### Protein identification using LC/MS/MS

To identify the extracellular protein secreted from *P. koreensis* HL12, the culture supernatant was precipitated in 85% (v/v) cold acetone on ice for 30 min; subsequently, centrifuged at 8,000 × *g* at 4 °C for 30 min. The protein pellet was rinsed and resuspended with 50 mM sodium phosphate buffer pH 7.4 and kept at 4 °C for 24 h. The prepared protein sample was digested into peptides using trypsin at 37 °C before it was injected into liquid chromatography with tandem mass spectrometry (LC–MS/MS) using TripleTOF 6600 + (Sciex, USA). The protein profile was identified by relating tandem mass spectra to the extracted database for *P. koreensis* (formerly known as *Bacillus koreensis*) from the NCBI database using PEAKS Studio 10.6. The identified protein that correlated with starch-degrading enzymes was aligned in BLASP (https://blast.ncbi.nlm.nih.gov/, accessed on 10 February 2023).

### Non-thermal saccharification of cassava pulp

The amylolytic activity against raw cassava pulp of the secreted enzyme from *P. koreensis* HL12 induced by raw cassava pulp was evaluated by measuring reducing sugar liberated from hydrolysate and then calculated conversion yield. The high solid loading of raw cassava pulp was performed at 5% (w/v) of substrate dissolved in 50 mM sodium citrate buffer pH 5.0. The enzymatic reactions with varying enzyme dosages of 1.25, 2.5, and 5.0 RSD U/g substrate were incubated at 45 °C, 200 rpm on an orbital shaker for 72 h. The hydrolysates were collected at 6, 12, 24, 48, and 72 h of incubation time for product analysis. The collected samples were inactivated by boiling for 10 min.

The obtained products from raw and gelatinized starch hydrolyses were investigated by reducing sugar measurement and product profile analysis. Spectrophotometric determination of amylase activity was conducted according to the DNS method (Miller 1959). The amount of reducing sugar was calculated from the measured absorbance at 540 nm. The conversion yield was calculated based on the amount of glucose produced relative to the initiation substrate.

$$\% {\text{Conversion}}\; {\text{yield}} = \frac{{{\text{Reducing}} \;{\text{sugar}}\; {\text{yield}} ({\text{mg}}/{\text{mL}})}}{{{\text{Initiation}}\; {\text{weight}} \;{\text{of}}\; {\text{biomass}} ({\text{mg}}/{\text{mL}})}} \times 100$$

### Product profile analysis using TLC

Product profile analysis was performed using thin-layer chromatography (TLC) compared with standard sugars, including glucose, maltose (G2), maltotriose (G3), maltotetraose (G4), maltopentaose (G5), and maltohexaose (G6). The released product profile from starch saccharification was analyzed using TLC Silica gel 60 F_254_ (Merck, Darmstadt, Germany) as a stationary phase. The TLC plate was spotted with the obtained products of hydrolysate and then immersed in a TLC chamber containing a mixture of mobile phase, n-butanol, acetic acid, and distilled water at a ratio of 5:2:3. After sufficient migration of the mobile phase, the TLC plate was thoroughly sprayed with a visualization solution consisting of a mixture of 95% (v/v) absolute ethanol and 5% (v/v) sulfuric acid modified by adding 0.1% (w/v) orcinol. The TLC plate was dried and heated until the spots appeared.

## Results

### Carbohydrate active enzymes (CAZymes) identified in *P. koreensis* HL12 genome

*P. koreensis* HL12 (formerly designated as *Bacillus koreensis*) was classified as an effective amylolytic-producing bacterium, which was isolated from soil close to sago palm root (*Cycas revoluta*), collected from Trang Province, Thailand. Based on 16S rDNA analysis and taxonomic classification based on genome sequence analysis using Tetra Correlation Search (TCS), the HL12 strain was identified as *P. koreensis* (Lekakarn et al. 2022, 2024). This strain demonstrated great potential for amylase production on screening medium, a minimal medium agar supplemented with 1% (w/v) soluble starch. Interestingly, *P. koreensis* HL12 presented effectively secreted amylase under cultivation in a minimal medium (0.5% (w/v) peptone and 0.5% (w/v) yeast extract). Therefore, this strain was studied with deep insight into amylolytic protein relevance.

In this work, genome sequencing analysis is the effective approach to elucidate genes related to the pathway involving starch decomposition and modification. Therefore, the whole genome sequence of *P. koreensis* HL12 was analyzed and has been deposited at DDBJ/ENA/GenBank under the accession JAUBKI000000000. The Clusters of Orthologous Genes (COGs) prediction by comparing the protein sequences of complete genomes based on databases of orthology relationships, functional annotation, and gene evolutionary histories revealed that carbohydrate transport and metabolism, amino acid transport and metabolism, inorganic transport, and metabolism and transcription were enriched COGs found in the genome.

Genome sequencing by the Illumina machine generated 4,204,469 pairs of raw reads with an average read length of 150 bps. The genome size of *P. koreensis* HL12 is 4,137,098 bps, comprising 180 assembled contigs with 304X genome coverage. The longest contig is 227,795 bps, with an N50 value of 67,414 bps. The GC content of the genome is 40.54%. The result from BUSCO indicates that the completeness of bacterial orthologous genes is 100%; meanwhile, the percentage of contamination calculated by CheckM against marker lineage of Bacillales is 0.57%. As demonstrated by the genome annotation using RAST, 4,529 genes were identified, including 4,425 protein-coding genes, 90 tRNA genes, 8 5S rRNA genes, 3 16S rRNA genes, and 3 23S rRNA genes. This information and all other relevant assembled and annotation statistics are in Table 1.

**Table 1:** Genome sequencing statistics

*Raw reads*	
# reads (PE)	4,204,469
X coverage	304X
*Genome assembly*	
Total length	4,137,098
# total contigs	180
# contigs (> = 10,000 bp)	76
# contigs (> = 50,000 bp)	18
Largest contig	227,795
N50	67,414
L50	14
GC (%)	40.54
# N's	327
# N's per 100 kbp	7.9
*Assessment of genome completeness and contamination by CheckM*	
Completeness	99.43%
Contamination	0.57%
*Assessment of gene completeness by BUSCO (bacteria_odb10)*	
Complete and single-copy BUSCOs (S)	124 (100%)
Complete and duplicated BUSCOs (D)	0
Fragmented BUSCOs (F)	0
Missing BUSCOs (M)	0
*Genome annotation by RAST*	
# Predicted gene	4,529
# Protein-coding gene	4,425
tRNA	90
5S rRNA	8
16S rRNA	3
23S rRNA	3

**Fig. 1:**
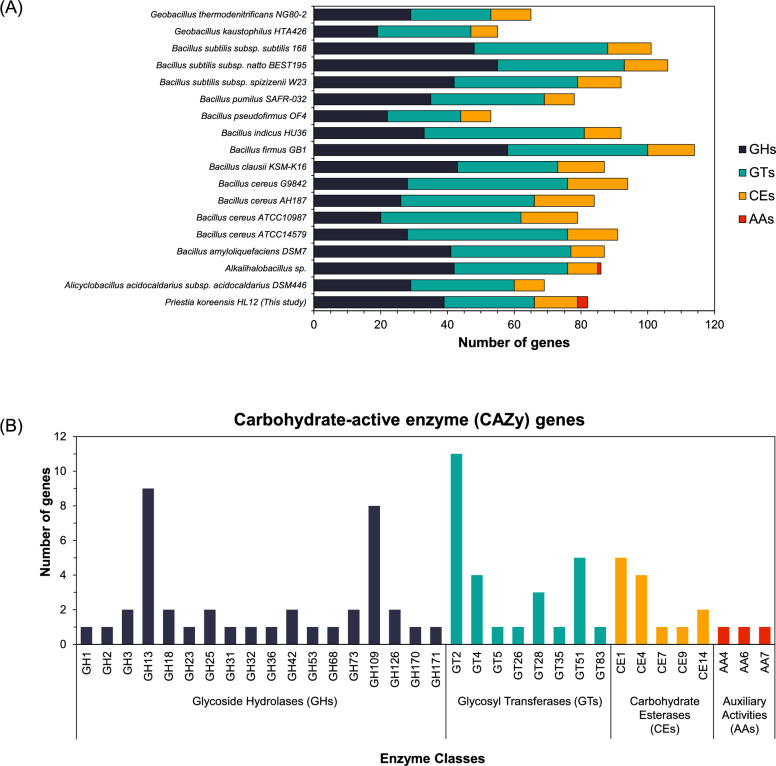
Genome sequence analysis **A** Comparative analysis of the number of putative genes for the five CAZyme categories in *Bacilli.* GH: Glycoside Hydrolases; GT: Glycosyl Transferases; CE: Carbohydrate Esterases; AA: Auxiliary Activities **B** Frequency of unique sequences of carbohydrate-active enzyme genes found in *P. koreensis* HL12 genome classified based on enzyme classes and families

Genes in the *P. koreensis* HL12 genome involved in carbohydrate metabolism were analyzed based on sequence annotation in the Carbohydrate-Active enZymes (CAZymes) database (http://www.cazy.org/). The result revealed that 82 unique sequences related to four CAZymes classes with numerous families were detected according to four families of carbohydrate-active enzymes: (i) Glycoside Hydrolases (GHs), (ii) Glycosyl Transferases (GTs), (iii) Carbohydrate Esterases (CEs), and (iv) Auxiliary Activities (AAs). Putative CAZymes in the *P. koreensis* HL12 genome are diverse in the same trend compared to various *Bacilli* (Fig. 1A). Most of them were categorized into the glycoside hydrolase family (39 numbers), followed by glycosyl transferase (27 numbers), carbohydrate esterases (13 numbers), and auxiliary activities (3 numbers), respectively (Fig. 1B). Focusing on each family, among the GH family, which is highly presented as GH13 and GH109 with 9 and 8 numbers, respectively, GH13 acts on substrates containing α-glucoside linkages, while GH109 members display an unusual mechanism involving NAD^+^. For glycosyl transferases (GTs), family GT2 is the most distinguished (10 numbers), followed by GT51 and GT54, which are close together (4 and 3 members). Most carbohydrate esterases (CEs) were categorized into CE1 and CE4 families. On the other hand, auxiliary activities (AAs) were found in each family consisting of AA4, AA6, and AA7. Most of these proteins are involved in the decomposition and modification of starch (α-1,4 and α-1,6-D-glucan), fructan (β-2,1 and β-2,6-D-fructan), and chitin (β-1,4-N-acetylglucosamine units). Not only glycoside hydrolases but also esterases, transferases, and auxiliary activities were found, including acetyl xylan esterases (CE1 and CE4), cellulose synthase (GT2), sucrose synthase (GT4), and glucooligosaccharide oxidase (AA7). In addition, according to genome sequence analysis, *P. koreensis* HL12 did not contain lignocellulosic genes encoding for pectinase and cellulase but found only one gene encoding for xylanase. These results defined *P. koreensis* HL12 as an amylolytic bacteria with specific starch-converting enzymes. Interestingly, various genes encoded for special enzymes were found in its genome, such as debranching type I pullulanase, isomaltulose synthase, maltodextrin glucosidase, and neopullulanase.

**Fig. 2:**
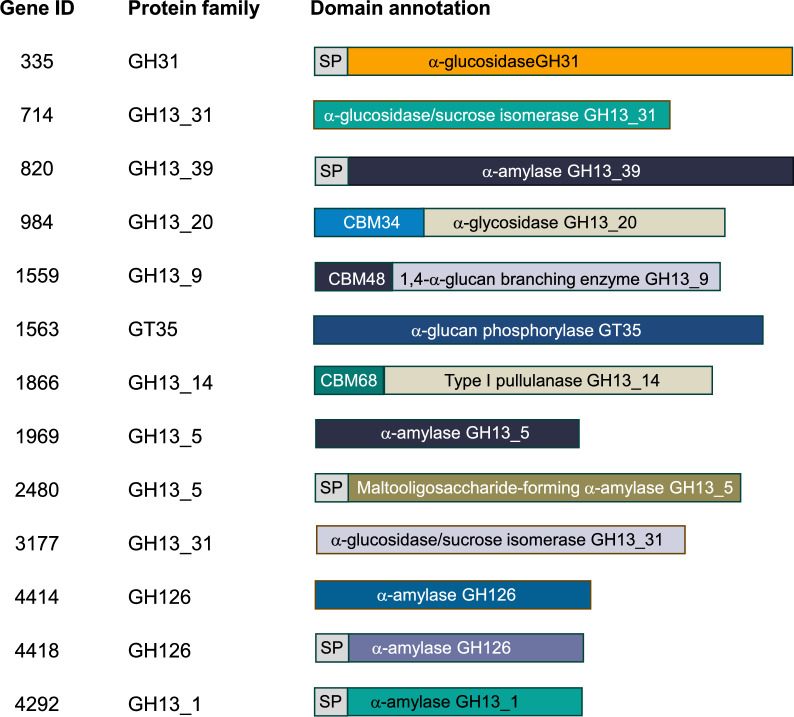
List of genes encoding starch degrading and modification enzymes available *P. koreensis* HL12 genome

Focusing on relevant starch-degrading enzymes, including GH3, GH13, GH14, GH15, GH31, GH57, GH119, and GH126, along with AA13 and GT35 (Møller et al. 2012; Møller and Svensson 2016), 13 unique sequences were obtained (Fig. 2). The majority of identified proteins were classified into GH13 (9 sequences) with various multi-domain architectures, followed by GH126 (2 sequences), GH31 (1 sequence), and GT35 (1 sequence), representing endo- and exo-acting proteins specific to α-1,4 and α-1,6 glycosidic linkages and transferase modes of action. The GH13 family contained various hydrolases that acted on α-1,4 and α-1,6-glycosidic bonds, for example, α-amylases (GH13_1, GH13_5, and GH13_39) and type I pullulanase (GH13_14), contributing to the decomposition of starch. Furthermore, cyclomaltodextrinases/malto-oligosyltrehalose synthase (GH13_20) and α-glucosidase/sucrose isomerases (GH13_31) responsible for starch modification were also identified in the *P. koreensis* HL12 genome. Importantly, glycogen/starch/α-glucan phosphorylase (GT35) that catalyzes the glycosidic linkages phosphorolysis to non-natural oligosaccharides was identified. These, therefore, demonstrated that the *P. koreensis* HL12 genome contained a set of genes necessary for the decomposition of starch and chemical structure modification.

### The effect of carbon sources on *P. koreensis* HL12 cell growth

**Fig. 3:**
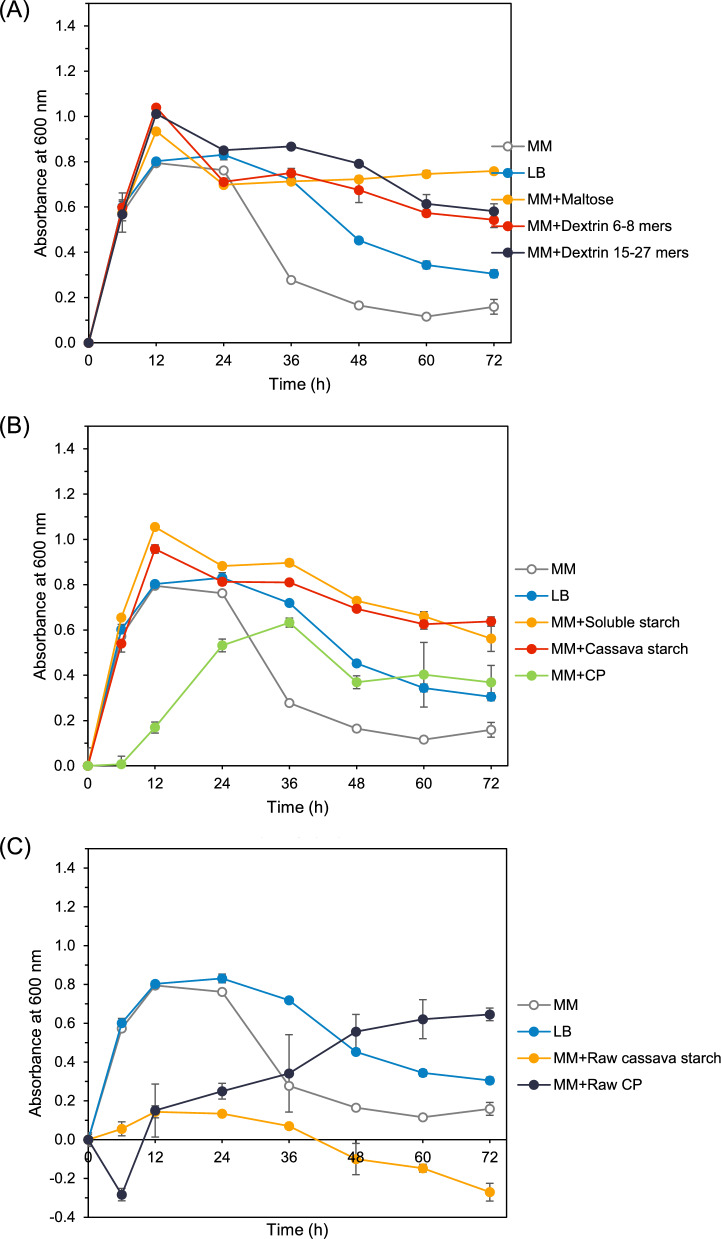
Effect of carbon sources on growth of *P. koreensis* HL12. Cell growth in minimal medium supplemented with various carbohydrate-inducers. **A** short-chain maltooligosaccharides (2–27 mers), **B** gelatinized starches and cassava pulp, **C** raw cassava starch and cassava pulp

The highly effective amylolytic bacterium, *P. koreensis* HL12, can grow in minimal medium, which provides the essential nutrients for the growth of wild strains. In this study, the minimal medium contained two nitrogen sources: peptone and yeast extract. However, varying the carbon sources used as inducers is an interesting strategy for enhancing the production of high-efficiency starch-degrading enzymes. In the minimal medium, *P. koreensis* HL12 exhibited a typical growth pattern, showing an exponential growth phase in 12 h, followed by a decline in cell growth after 24 h. This pattern was similar to the minimal medium supplemented with maltooligosaccharides and gelatinized starch. The maximum growth of *P. koreensis* HL12 was observed in a minimal medium supplemented with gelatinized soluble starch. In contrast, minimal media supplemented with raw starches resulted in delayed cell growth. Interestingly, while cell growth was initially slow, it progressively increased over 72 h of incubation in a minimal medium supplemented with raw cassava pulp (Fig. 3A–C).

### The effect of carbohydrate inducers on amylolytic activity of the secreted enzymes from *P. koreensis* HL12

**Fig. 4:**
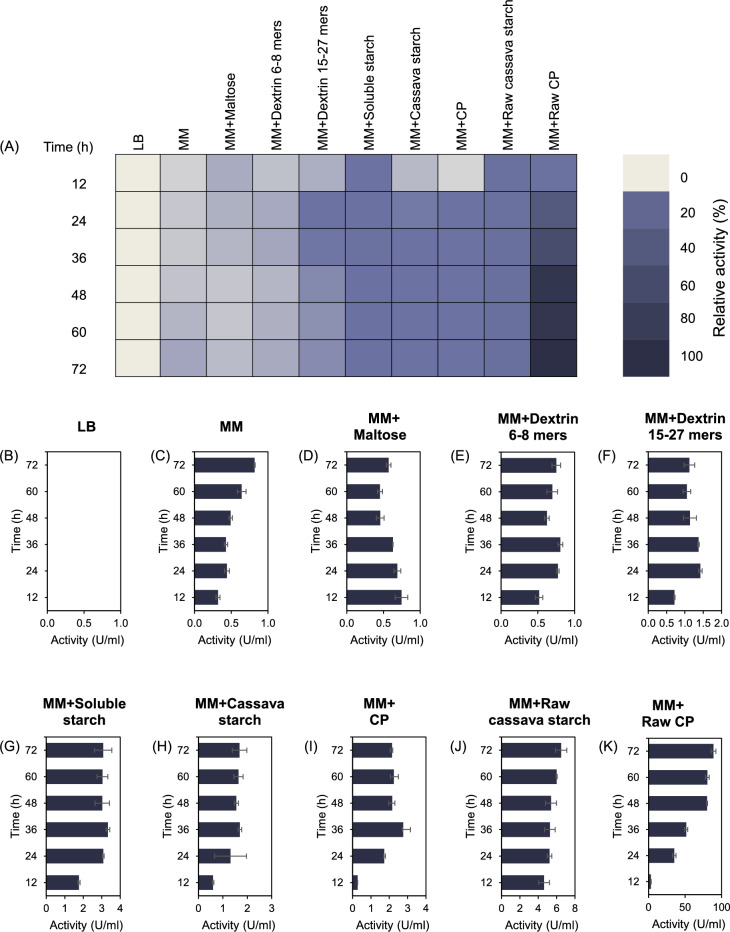
The amylolytic activity of the secreted enzymes from *P. koreensis* HL12 cultured in medium supplemented with various carbohydrate-inducers. **A** Relative activity of secreted starch degrading enzyme from *P. koreensis* HL12 cultivated in different carbon sources showing as heat map. **B**–**K** Amylase activity of the secreted enzymes produced from the medium supplemented with various carbohydrate-inducers

To evaluate the effect of carbohydrate inducers on amylolytic activity of the extracellular enzymes, enzyme activity of the secreted fraction in media supplemented with different inducers was analyzed using the DNS method. For quantitative analysis of the amylase activity of the secreted enzyme using different carbohydrate inducers, the starch-degrading activity was evaluated using 1% (w/v) cassava starch as substrate (Fig. 4). Under a defined condition (50 mM sodium acetate buffer, pH 5.0, 50 °C, for 10 min), the secreted protein using raw cassava pulp as a carbon source exhibited remarkable starch-degrading activity when compared to another medium (Fig. 4A). In contrast, secreted protein from LB broth did not show starch-degrading activity (Fig. 4B). Different chain lengths of oligosaccharides used as carbohydrate inducers induced more amylolytic enzyme activity with the larger chain length than short-chain oligosaccharides, including maltose and maltodextrin 6–8 oligomers (Fig. 4D-F). Various gelatinized and raw starches could significantly increase the secretion of starch-degrading enzymes compared to a minimal medium without a carbon source (Fig. 4C, G-K). The maximum starch-degrading activity was observed when using raw cassava pulp as an inducer in minimal medium with specific activity 452.6 ± 2.944 U/mg protein against cassava starch under a defined condition (Fig. 4K).

According to amylolytic analysis through zymography technique and enzymatic assay, MM media supplemented with raw cassava pulp can induce the secretion of starch-degrading enzyme from *P. koreensis* HL12 with the highest activity, indicating that raw cassava pulp is a potential inducer for amylolytic enzyme production. Therefore, the optimal working condition of the secreted protein was further determined, and relevant amylolytic enzymes were subsequently identified using gel-free base proteomic approach.

### Exploration of optimal working conditions for the secreted starch-degrading enzyme induced using raw cassava pulp (HL12RCP)

**Fig. 5:**
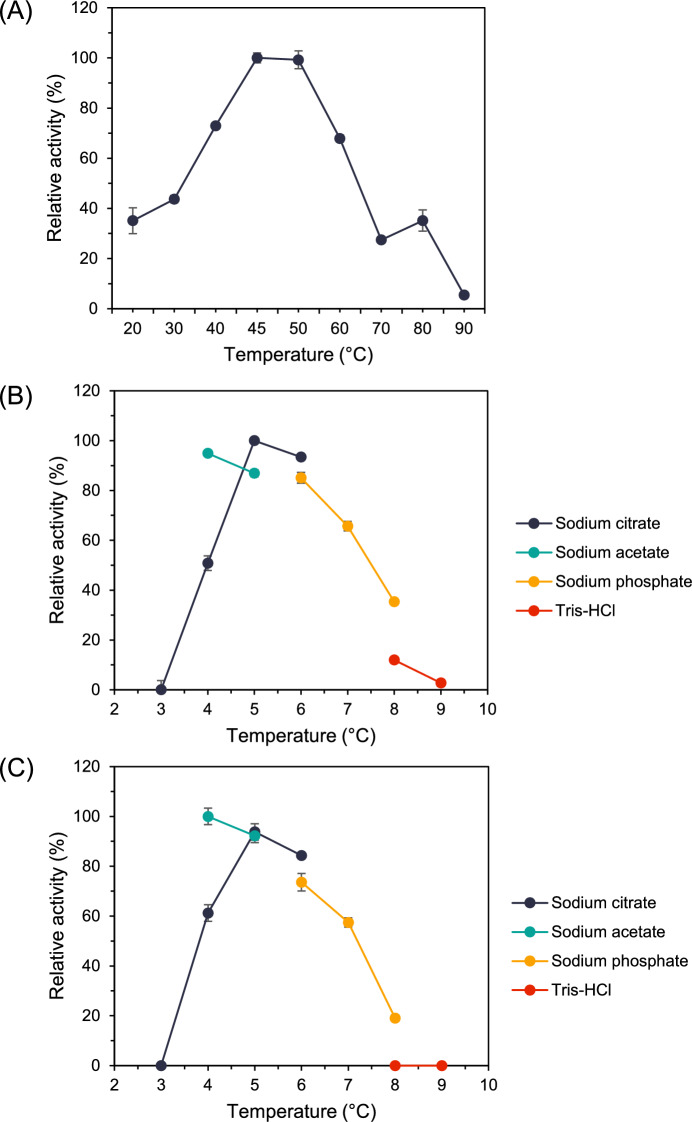
The effect of temperature and pH on amylase activity of HL12RCP. **A** The effect of temperature ranging from 30 to 80 °C **B** The effect of pH was measured in various buffers ranging from pH 3.0–9.0 at 45 °C and **C** the effect of pH was measured in various buffers ranging from pH 3.0–9.0 at 30 °C

Under the standard condition, the secreted-degrading enzyme induced using raw cassava pulp (HL12RCP) exhibited highly effective amylolytic enzyme with specificity 627.2 ± 8.708 U/mg protein and 402.7 ± 4.781 U/mg protein toward gelatinized soluble starch and cassava starch, respectively. In order to define optimal conditions for starch-degrading activity, the effects of temperature and pH were investigated. According to the effect of temperature, HL12RCP was determined under temperatures ranging from 20 to 90 °C using gelatinized cassava starch as substrate. HL12RCP exhibited maximum activity at 45 °C and retained more than 60% relative activity at 40–60 °C, indicating that HL12RCP prefers working at moderate temperatures. Interestingly, HL12RCP has two ranges of working temperature, which were optimal at 45–50 °C and declined until 70 °C. After that, starch-degrading activity slightly increased at 80 °C, suggesting that HL12RCP comprises at least two major groups of amylolytic enzymes with different working temperatures preferences (Fig. 5A).

Regarding the development of the starch saccharification under ambient temperature, the effect of pH was evaluated with varying pH ranges of 3.0–9.0 at 45 and 30 °C. Under 45 °C, HL12RCP provided the highest starch-degrading activity in 50 mM sodium citrate buffer pH 5.0. Remarkably, more than 80% remaining activity was observed in 50 mM sodium acetate buffer pH 4.0 and sodium phosphate buffer pH 6.0. However, starch-degrading activity retained more than 50% relative activity in 50 mM sodium citrate buffer pH 4 and sodium phosphate buffer pH 7.0, whereas dramatically decreased at pH 8.0–9.0 (Fig. 5B). Likewise, the pattern of effect of pH at 30 °C was similar to that of 45 °C. However, the maximum activity was presented in 50 mM sodium acetate buffer pH 4.0 (Fig. 5C). These results showed that HL12RCP could hydrolyze starch at broad range conditions in both temperature and pH with high activity; consequently, HL12RCP is considered a potential secreted enzyme for starch saccharification application. For cassava starch saccharification, HL12RCP showed effective amylolytic activity with specific activity 430.1 ± 10.90 U/mg protein toward cassava starch at optimal working conditions (sodium citrate buffer pH 5.0, 45 °C) with no detectability toward xylanase, pectinase, and cellulase activity. Regarding the application of HL12RCP in raw starch hydrolysis, the starch-degrading activity of HL12RCP toward raw cassava pulp was determined under the optimal condition, which provided raw starch degrading (RSD)-specific activity with 2.238 ± 0.130 RSD U/mg protein.

### Proteomic and genomic analysis of starch-converting enzymes relevant

The amylolytic activity of the extracellular crude enzyme produced by *P. koreensis* HL12 was evaluated in relation to various polysaccharides that served as carbon sources and inducers. Notably, the highest enzymatic activity was observed in the minimal medium supplemented with raw cassava pulp. To identify proteins associated with starch degradation and modification, extracellular proteins secreted by *P. koreensis* HL12 cultured in this medium (designated H12RCP) were analyzed using liquid chromatography-tandem mass spectrometry (LC/MS/MS). The analysis detected a total of 86 proteins within the precipitated extracellular protein fraction (Additional file 1: Table S1). Among these, two proteins directly related to starch hydrolyzing and modification were identified: 1 α-amylase (gene ID 2480) and 1 oligo-1,6-glucosidase (gene ID 3177). Importantly, no corresponding proteins were detected in the negative control, which consisted of the minimal medium supplemented with raw cassava pulp. This confirmed that the identified proteins were specifically produced by *P. koreensis* HL12.

Comprehensive sequence analysis of α-amylase (gene ID 2480) revealed that, based on its conserved domain, it is classified within glycoside hydrolase family 13, subfamily 5 (GH13_5) in the α-amylase superfamily (cd11318). This classification suggests that it may exhibit activities characteristic of maltopentaose-producing α-amylase (EC 3.2.1.-), α-amylase (EC 3.2.1.1), maltotriose-producing α-amylase (EC 3.2.1.116), or maltohexaose-producing α-amylase (EC 3.2.1.98). In the case of oligo-1,6-glucosidase (gene ID 3177), its conserved domain corresponds to the α-amylase catalytic domain (cd11333). This domain is also found in sucrose isomerases, oligo-1,6-glucosidase (often referred to as isomaltose; sucrase-isomaltase; α-limit dextrinase), dextran glucosidase (also known as glucan 1,6-α-glucosidase), and other related proteins. In terms of GH family classification, the enzyme was categorized into the GH13_31 subfamily, which encompasses activities such as oligosaccharide α-4-glucosyltransferase (EC 2.4.1.161), palatinase (EC 3.2.1.-), α-amylase (EC 3.2.1.1), oligo-α-1,6-glucosidase (EC 3.2.1.10), α-glucosidase (EC 3.2.1.20), glucodextranase (EC 3.2.1.70), and isomaltulose synthase/sucrose isomerase/sucrose glucosylmutase (EC 5.4.99.11) activities. Previous studies have shown that an α-glucosidase protein classified within GH13_31 subfamily, isolated from *Bacillus* sp. AHU2216, exhibited high transglucosylation activity, with a strong preference for maltose (DP2) (Auiewiriyanukul et al. 2018).

### Potential applications of HL12RCP for upcycling high-solid loads from cassava processing waste

**Fig. 6:**
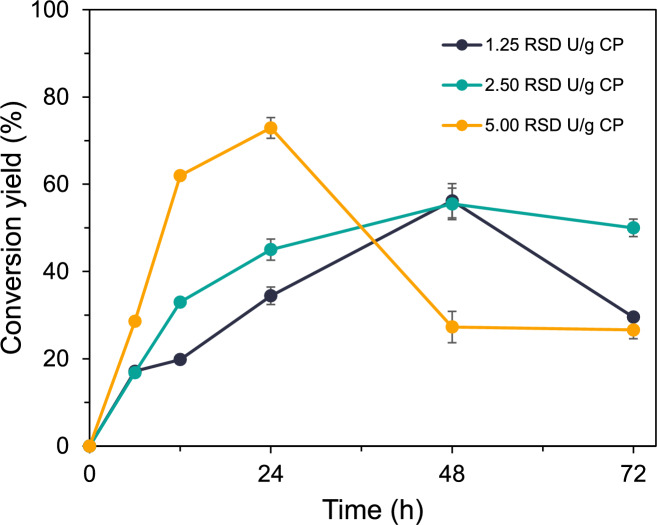
The effect of enzyme loading of HL12RCP on raw cassava starch saccharification. The reaction contained 5% (w/v) raw cassava starch in 50 mM sodium citrate buffer pH 5.0 with varying enzyme dosage. The liberated reducing sugars were measured using the DNS method. The diagram presented % conversion yield from starch saccharification

To promote starch-based biorefineries through the upcycling of starch-containing waste, cassava pulp was evaluated as a feedstock. Based on amylolytic activity and supported by proteomic and genomic analyses, the crude enzyme secreted by *P. koreensis* HL12, cultured in a minimal medium supplemented with raw cassava pulp (designated HL12RCP), was utilized as a biocatalyst for the saccharification of raw cassava pulp. To focus on high solid substrate loading, 5% (w/v) raw cassava pulp was used as the substrate, with varying different enzyme dosages. The highest loading of HL12RCP (5 RSD U/g substrate) resulted in a maximum reducing sugar production yield of 719.1 mg/g biomass, corresponding to a conversion yield of 72.12% ± 3.975 after 24 h of hydrolysis. Notably, a 12-h hydrolysis period yielded a conversion exceeding 50%. Additionally, the maximum reducing sugar yields from enzyme dosages of 1.25 and 2.5 RSD U/g biomass after 48 h were 566.5 ± 18.19 mg/g biomass and 565.6 ± 52.31 mg/g biomass, which equated to conversion yield of 55.32% ± 3.478 and 55.24% ± 2.484, respectively (Fig. 6). Interestingly, after 48 h of incubation at the highest enzyme dosage (5.0 RSD U/g substrate), the amount of sugar products obtained decreased. This observation aligns with findings from genomic and proteomic analyses, which indicate that the extracellular crude HL12RCP contains a mixture of various enzymes with different catalytic activities and biochemical characteristics. The genome of *P. koreensis* HL12 genome revealed multiple starch-converting enzyme genes, some of which are classified as glycosyltransferase, capable of generating larger molecules from smaller sugars, such as maltodextrin and cyclodextrin. This phenomenon is consistent with the presence of glycosyltransferase genes in the *P. koreensis* HL12 genome.

**Fig. 7:**
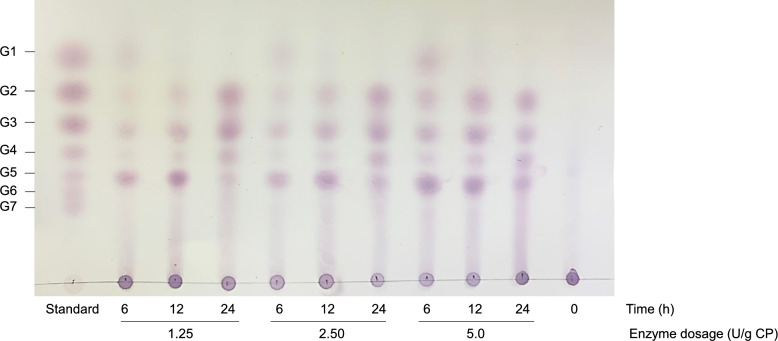
The effect of enzyme loading and incubation times on product profiles of raw cassava pulp saccharification by HL12RCP. Product profiles were analyzed using TLC technique compared to standard. G1, G2, G3, G4, G5, and G6 represent glucose, maltose, maltotriose, maltotetraose, maltopentaose, and maltohexaose. Blank represents 5% (w/v) of raw cassava pulp without enzyme

The obtained product was analyzed to determine the pattern of product profile and product specificity from the hydrolysis of cassava pulp using thin layer chromatography (Fig. 7). After six hours of hydrolysis, maltotriose (G3) and maltopentaose (G5) were liberated as the major products, followed by glucose (G1), maltose (G2), and maltotetraose (G4). The concentration of these products increased with the rising enzyme dosage. As the incubation time extended, the levels of short-chain oligosaccharides (G2-G5) increased, while glucose levels decreased and eventually vanished. This reduction in glucose and the short-chain oligosaccharides in the product profile was consistent with proteomic analysis, which identified several carbohydrate-modifying enzymes, including glycosyltransferase, in HL12RCP. These enzymes are known to catalyze the formation of larger molecules from smaller sugar.

## Discussion

Due to its high capacity for amylolytic enzyme production, *P. koreensis* HL12 represents a promising novel source of enzymes for the saccharification and modification of starch-rich materials. The putative CAZymes in the genome of *P. koreensis* HL12 were categorized and compared to various *Bacilli*. Data derived from Fig. 1A present a comparative analysis of the number of putative genes across the five CAZyme categories, providing insights into the distribution of these enzymatic functions among *Bacilli*. (Manzo et al. 2011; Masasa et al. 2022). A total of 82 CAZymes were identified in the *P. koreensis* HL12 genome, comprising 39 genes encoding GHs, 27 genes encoding GTs, 13 genes encoding CEs, and 3 genes encoding AAs. Although *P. koreensis* HL12 does not have the highest number of CAZymes among *Bacilli*, it is notable for its significant representation of GHs as a major group. Specifically, *P. koreensis* HL12 possesses a large number of CAZymes from GH13 and GT2 families, as well as several CAZyme families that are not shared with other *Bacilli*, particularly GH109, GT53, GH126, GH170, and GH171. Additionally, it includes CAZymes from families GH25, GH31, GH36, GH53, and GH68 (Manzo et al. 2011).

*P. koreensis* HL12, a promising producer of carbohydrate-active enzymes (CAZymes), can grow in a peptone-based medium without added sugars or carbon sources. However, to enhance the production yield and explore novel amylases, the optimization of carbon sources was evaluated. Various types and forms of carbon sources were tested, ranging from simple sugars (short-chain oligosaccharides) to different forms of starch, both gelatinized and raw forms and complex structures such as cassava pulp. The highest amylolytic activity was observed when raw cassava pulp was used as a carbon source. Cassava pulp is widely recognized as a feedstock for biorefineries and has been utilized as a raw material for decades (Bunterngsook et al. 2017; Djuma’ali et al. 2011; Lerdlattaporn et al. 2021; Sudha et al. 2015; Valeriano et al. 2018; Virunanon et al. 2013). However, there are few reports on its use as a carbon source for inducing amylase production. For example, Diaz et al. (2020) noted that *Aspergillus niger* LBM 134, when grown on cassava bagasse, produced lower specific amylase yields compared to *P. koreensis* HL12. While *A. niger* LBM 134 secreted a mixture of amylase, cellulase, and pectinase (Diaz et al. 2020), *P. koreensis* HL12 exclusively produced starch-degrading enzymes, as indicated by genomic analysis that showed no genes for cellulose or fiber-degrading enzymes. Typically, wild strains produce extracellular enzymes as a cocktail containing various enzyme types, which can limit their effectiveness in agro-biomass conversion due to low specificity and product purity. In contrast, the enzymes secreted by *P. koreensis* HL12 in the presence of cassava pulp are highly specific for starch-containing materials. Although cassava pulp contains both starch and lignocellulosic fibers, the final product yielded high purity maltooligosaccharides derived from starch. These results indicate that *P. koreensis* HL12 can be an effective microbial strain for precision fermentation in starch-based biorefinery applications (Augustin et al. 2024; Siddiqui et al. 2023). Experiments involving various chain lengths of maltooligosaccharides, maltose, and maltodextrins (6–8 and 15–27 oligomers) showed lower amylase activity compared to more complex substrates. Various *Bacilli* possess transporters for maltooligosaccharides, as well as cyclodextrin and maltodextrin ATP-binding cassette transporters, which facilitate the uptake of maltooligosaccharides into cells (Kamionka and Dahl 2001; Nakai et al. 2009; Schönert et al. 2006; Tangney et al. 1992). Consequently, these transporters reduce the need for the production of large quantities of enzymes to degrade complex carbon sources into smaller sugars, leading to lower expression of secreted amylolytic enzymes.

The substantial amount of reducing sugar indicates the potential for high-efficiency hydrolysis of raw cassava pulp with significant substrate loading. This suggests that starch saccharification, using moderately enzymatically treated cassava pulp, could serve as a cost-effective substrate for maltooligosaccharide production. Regarding starch-based MOS production, the hydrolytic potential of the starch-degrading enzyme secreted by *P. koreensis* HL12 does not require thermal treatment or pretreatment processes, unlike conventional enzymatic and chemical methods that necessitate high-temperature processes for starch gelatinization and liquefaction (Djuma’ali et al. 2011; Sudha et al. 2015; Virunanon et al. 2013). This highlights *P. koreensis* HL12 as a candidate for an amylolytic-producing cell factory capable of complete cassava pulp saccharification, as well as starchy by-products and food waste such as potato pomace, rice bran, wheat bran, and bread waste through a non-thermal enzymatic process to generate high value maltooligosaccharides.

Regarding the valorization of starch-rich by-products, cassava pulp (CP) has been successfully used as a feedstock for specific starch-degrading enzymes and maltooligosaccharide (MOS) production through batch fermentation of *P. koreensis* HL12 at the flask scale. This demonstrates the potential of fermentation technology in converting starch processing by-products into high-value products aligning with Sustainable Development Goals (SDGs) and the bio-circular green economy while enhancing food security and environmental sustainability (Siddiqui et al. 2023). The saccharification of cassava pulp using HL12RCP treatment exhibited high starch-hydrolytic activity, generating short-chain maltooligosaccharide (G1–G5). This corresponds to proteomic analysis indicating that maltooligosaccharide-forming amylase (EC 3.2.1.98) is the primary enzyme responsible for the hydrolytic activity observed in the secreted enzyme from *P. koreensis* HL12 induced with raw cassava pulp. This discovery reveals the effectiveness of *P. koreensis* HL12 as a promising source for amylase production aimed at maltooligosaccharides (Monma et al. 1983; Nakakuki et al. 1983). Interestingly, glucose, a monosaccharide present in the 6 h H12RCP treatment, dramatically decreased over longer incubation times, corresponding with a reduction in the total amount of reducing sugars. Genome and proteomic analyses of *P. koreensis* HL12 suggest that glucose and/or small maltooligosaccharides may be converted into larger molecules by glycosyltransferases (EC 2.4.1.1), which catalyze the formation of glycosidic bonds from an activated donor sugar onto another saccharide (Breton et al. 2005; Lairson et al. 2008). Notably, Monma et al. (1983) reported that a glycosyltransferase in exo-maltohexaohydrolase binds G2 at the catalytic site and transfers it to the non-reducing end of G4 to release G6 as a product, demonstrating substrate concentration-dependent action. These findings support the observed loss of small-size reducing sugars during the product analysis of raw cassava pulp saccharification by HL12RCP. This highlights the high potential for complete raw cassava starch saccharification using starch-rich agricultural by-product. The synergistic effect between hydrolytic and modifying enzymes from *P. koreensis* HL12 produces high-value short-chain maltooligosaccharides without generating glucose by-products, reducing the need for extensive purification. However, identifying the optimal conditions for maltooligosaccharide synthesis during bioconversion remains a key challenge in this research. Precision fermentation combined with effective process design will facilitate the simultaneous synthesis of both enzymes and MOS in a single pot. Furthermore, an in-depth understanding of the biochemical properties of the enzymes secreted by *P. koreensis* HL12, as revealed through proteomic analysis, will provide critical insights to improve control over fermentation and product synthesis. This study significantly advances sustainable starch-based biorefineries by focusing on the discovery of efficient starch-degrading and converting enzymes from *P. koreensis* HL12. By utilizing starch-rich agricultural by-products such as cassava pulp, it promotes resources valorization to produce high-value products, enhancing enzyme production and supporting the growth of starch-based biorefineries.

## Conclusion

In summary, the integration of multi-omic approaches and enzymatic profiling conducted in this study provides valuable insights essential for the systematic optimization of enzyme production. This optimization is crucial for the design of effective enzymes tailored for the saccharification of starch-rich biomass to yield high-value functional products. The results underscore that *P. koreensis* HL12 has significant potential as an efficient amylase producer when utilizing cassava pulp as feedstock for enzyme production and maltooligosaccharide synthesis. This finding opens avenues for developing starch-based biorefineries that employ moderate enzymatic processing, highlighting the feasibility and sustainability of using cassava pulp in bioconversion processes.

## Supplementary Information

Additional file 1.

## Data Availability

Data will be made available on request.
