# Supplementary material for: Boosting secretion of starch-converting enzymes from Priestia koreensis HL12 and its application in non-thermal cassava pulp saccharification process for maltooligosaccharides synthesis
Source: Bioresour Bioprocess. 2025 Apr 21;12(1):37. doi: 10.1186/s40643-025-00872-x (PMC12011666; doi:10.1186/s40643-025-00872-x)
Supplement: Supplementary file 1 — Additional file 1. [file 40643_2025_872_MOESM1_ESM.pdf]

Supplementary materials

Boosting secretion of starch-converting enzymes from *Priestia koreensis* HL12 and its application in non-thermal cassava pulp saccharification process for maltooligosaccharides synthesis

Daran Prongjit[1], Benjarat Bunterngsook[2], Wuttichai Mhuantong[2], Katesuda Aiewviriyasakul[2],
Wipawee Sritusnee[2], and Hataikarn Lekakarn[1,*]

[1]Department of Biotechnology, Faculty of Science and Technology, Thammasat University, Rangsit Campus, Khlong Nueang, Khlong Luang, Pathum Thani 12120, Thailand
[2]Enzyme Technology Research Team, Biorefinery Technology and Bioproduct Research Group, National Center for Genetic Engineering and Biotechnology, 113 Thailand Science Park, Phahonyothin Road, Khlong Nueang, Khlong Luang, Pathum Thani 12120, Thailand

*Corresponding author. Address: Department of Biotechnology, Faculty of Science and Technology, Thammasat University, Rangsit Campus, Khlong Nueang, Khlong Luang, Pathum Thani 12120, Thailand

Tel.: +662-564-4441 (ext. 2452)

E-mail address: hataikarn.lek@sci.tu.ac.th (Hataikarn Lekakarn)

ORCID ID: 0000-0001-9383-5098

Table S1: List of proteins detected from concentrated extracellular protein (HL12RCP) secreted from *P. koreensis* HL12 cultured in MM supplemented raw cassava pulp medium.

Description	Average Mass (Da)
Aconitate hydratase	98999
Flagellin	54283
Aminopeptidase	41830
ABC transporter substrate-binding protein	62635
Betaine-aldehyde dehydrogenase	55047
Alpha-amylase	58349
Uncharacterized protein	132229
Metal-dependent carboxypeptidase	58487
DNA-binding protein	55975
Oligoendopeptidase F	69836
ABC transporter substrate-binding protein	66692
Oligoendopeptidase F	65560
2,' 3'-cyclic nucleotide 2'-phosphodiesterase	78692
ABC transporter substrate-binding protein	47642
Cytochrome C	60999
Leucine dehydrogenase	40257
Xaa-Pro dipeptidase	38730
Enolase	46443
M20_dimer domain-containing protein	39661
Flagellin	26843
Thioredoxin	11476
2', 3'-cyclic nucleotide 2'-phosphodiesterase	94912
Cytochrome D ubiquinol oxidase subunit I	51916
ABC transporter substrate-binding protein	46824
Arginase	32112
Ketol-acid reductoisomerase (NADP(+))	37287
Phosphopentomutase	44103
Catalase	55101
Uncharacterized protein	27872
Phosphate acetyltransferase	34631
S-ribosylhomocysteine lyase	17821
Putative septation protein SpoVG	10708
Uncharacterized protein	23901
Succinate dehydrogenase	66740
N-acetylmuramoyl-L-alanine amidase	26032
Dipeptidase PepV	51760
Glutamine amidotransferase	19619
Nucleoside diphosphate kinase	16559
Glutamine synthetase	50233
Oligo-1,6-glucosidase	65214

Description	Average Mass (Da)
Cytochrome c oxidase subunit 1	73372
Oxidoreductase	41195
Peptidase	74684
Tautomerase	6832
UDP-glucose 4-epimerase	36659
NH(3)-dependent NAD(+) synthetase	30889
Peptide ABC transporter substrate-binding protein	57358
L-lactate dehydrogenase	34942
2-oxoisovalerate dehydrogenase	35441
Phenylhydantoinase	52136
Esterase	50775
3,4-dihydroxyphenylacetate 2,3-dioxygenase	37656
PalA	27375
Quinol oxidase subunit 3	22808
Peptidyl-prolyl cis-trans isomerase	15789
NADH dehydrogenase	55038
Zinc protease	46515
Peptidase T	45551
Dipicolinate synthase subunit B	21987
Purine nucleoside phosphorylase	29153
Cytochrome c oxidase subunit 1	68787
Chemical-damaging agent resistance protein C	20721
NAD(P)H nitroreductase	22324
N-acetyltransferase domain-containing protein	46167
Phosphate-binding protein	31832
NonF	23649
NMT1 domain-containing protein	37853
Zinc transporter ZitB	33184
Purine nucleoside phosphorylase DeoD-type	25719
2,3-bisphosphoglycerate-independent phosphoglycerate mutase	56477
Uncharacterized protein	20388
NADPH-dependent oxidoreductase	28445
Superoxide dismutase	22431
Beta-galactosidase	76305
Cytochrome C biogenesis protein	43968
Chorismate mutase	39483
Uncharacterized protein	19167
Arsenic transporter	50751
Multifunctional fusion protein	82183
ABC transporter substrate-binding protein	45339
Polyamine aminopropyltransferase	31116
Cytochrome d ubiquinol oxidase subunit 2	37514
Hydantoinase	47236

Description	Average Mass (Da)
Uncharacterized protein	23704
Beta-galactosidase	78857
ESAT-6-like protein	10763

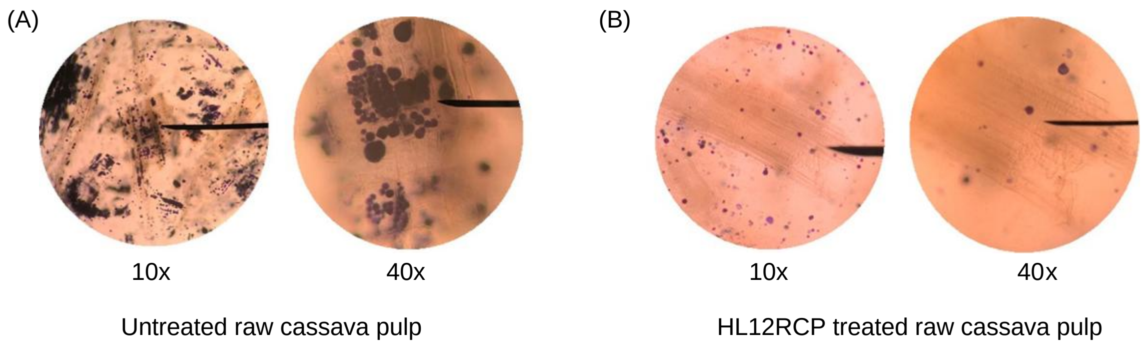

Fig. S1 The morphology of iodine-stained raw cassava pulp after hydrolysis 72 hours with 5 RSD U/g substrate under microscope with 10x and 40x magnification. (A) Untreated raw cassava pulp (B) Raw CP treated with 5 U/g substrate of HL12RCP for 72 h.
